# Genomic landscape of Waldenström's macroglobulinemia

**DOI:** 10.1097/HS9.0000000000000228

**Published:** 2019-06-30

**Authors:** Steven P. Treon, Zachary R. Hunter, Andrew R. Branagan, Jorge J. Castillo

**Affiliations:** 1 Bing Center for Waldenström's Macroglobulinemia, Dana-Farber Cancer Institute, Boston, United States; 2 Department of Medicine, Harvard Medical School, Boston, United States; 3 Division of Hematology-Oncology, Massachusetts General Hospital, Boston, United States

**Take home messages**

- Next-generation sequencing has revealed recurring somatic mutations in Waldenstrom's Macroglobulinemia that include MYD88 (95%–97%), CXCR4 (30%–40%), ARID1A (17%) and CD79B (8%–15%).
- Deletions involving chromosome 6q are common in MYD88 mutated patients, and include genes that modulate NFKB, BCL2, BTK, and apoptosis.
- Wild-type MYD88 Waldenström's Macroglobulinemia (WM) patients show an increased risk of transformation and death, and exhibit many mutations found in diffuse large B-cell lymphoma.
- Response to the BTK inhibitor ibrutinib is impacted by both MYD88 and CXCR4 mutation status in WM.

## Introduction

Whole genome sequencing has identified recurring somatic mutations in MYD88, CXCR4, ARID1A, and CD79, along with copy number alterations including those in chromosome 6q that impact regulatory genes affecting NFKB, BTK, BCL2, and apoptosis.^1^ Herein, we discuss the genomic landscape of Waldenström's Macroglobulinemia (WM), and the impact of underlying genomics on disease presentation, treatment outcome, and overall survival impact.

**Figure 1:**
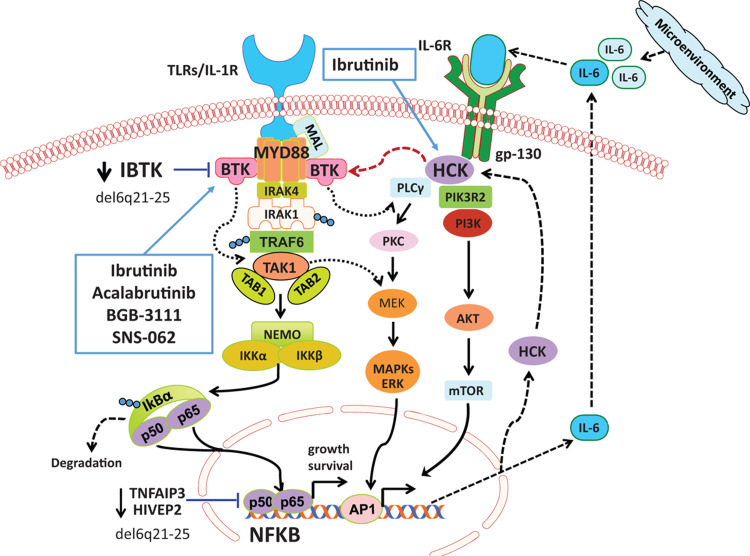
**Mutated MYD88 related signaling in Waldenstrom's Macroglobulinemia.** Mutated MYD88 transactivates NFKB through divergent pathways that include IRAK1/IRAK4 and BTK. Mutated MYD88 also triggers transcription and activation of the SRC family member HCK. Activated HCK can then trigger BTK, AKT, and ERK1/2 mediated pro-growth and survival signaling in WM cells. Copy number abnormalities including loss of 6q are common in WM patients, and impact regulators of MYD88 signaling including inhibitor of BTK (IBTK), TNFAIP3, and HIVEP2. Ibrutinib can target BTK and HCK.

## Current state of art

### Mutations in MYD88

A recurring somatic mutation in MYD88 (MYD88 L265P) was identified in 91% of WM patients by paired tumor/normal whole genome sequencing.^2▪^ By more sensitive allele-specific PCR testing, MYD88 L265P was expressed in 93% to 97% of WM patients. Non-L265P MYD88 mutations have also been identified in 1% to 2% of WM patients.^3^ MYD88 mutations are also detectable in 50% to 80% of cases of IgM Monoclonal Gammopathy of Unknown Significance (MGUS), suggesting an early oncogenic role for WM pathogenesis.^4^–^6^

Both IRAK1/IRAK4 and BTK are components of the MYD88 signaling complex, termed the “Myddosome” and trigger NFKB pro-survival signaling.^7^^,^^8▪^ Mutated MYD88 can also transcriptionally upregulate as well as transactivate HCK, a SRC family member that is normally downregulated in late stages of B-cell ontogeny.^9^ Activated HCK in turn triggers pro-survival signaling of mutated WM cells through BTK, PI3K/AKT and MAPK/ERK1/2. Both BTK and HCK are targets of ibrutinib, that has shown remarkable activity in MYD88 mutated WM patients.^9^

### Mutations in CXCR4

Mutations in the C-terminal domain of CXCR4 are present in up to 40% of WM patients when isolated bone marrow CD19-selected cells are used for sequencing, and while they almost always occur with MYD88 mutations, some patients with wild-type MYD88 can also express these mutations.^1^^,^^10▪^ CXCR4 mutations are essentially unique to WM, with only rare reports in marginal zone lymphoma (MZL) and ABC diffuse large B-cell lymphoma (DLBCL) cases.

Unlike MYD88, CXCR4 mutant clonality is highly variable. Multiple CXCR4 mutations can also exist within individual patients that occur in separate clones or are present as compound heterozygous events.^11^ The subclonal nature of CXCR4 mutations relative to MYD88 suggests that these mutations occur after MYD88, though this is likely to be an early event in WM pathogenesis given their detection in IgM MGUS patients. Clonal 6q deletions which are found in 40% to 50% of WM patients, appear exclusive of CXCR4.^12^

Patients with CXCR4 mutations present with a significantly lower rate of adenopathy, and those with CXCR4 nonsense mutations have increased bone marrow disease, serum IgM levels, and/or symptomatic hyperviscosity. Despite differences in clinical presentation, CXCR4 mutations do not appear to adversely impact overall survival in WM.^13^ In vitro modeling of WM cells transduced with mutated CXCR4 showed increased drug resistance in the presence of CXCL12 to multiple therapeutics including bendamustine, fludarabine, bortezomib, idelalisib, and ibrutinib.^14^,^15^

### Other recurring mutations

Somatic mutations in ARID1A are present in 17% of WM patients, nonsense and frameshift variants.^1^ Patients with ARID1A and MYD88 L265P mutations showed greater bone marrow disease involvement, and lower hemoglobin and platelet count. ARID1A and its frequently deleted homolog ARID1B (discussed below) are on chromosome 6q.^1^,^12^ Both serve as chromatin remodeling genes (CRG) thereby modulating gene regulation. While still poorly understood, ARID1A can modulate TP53, and is thought to act as an epigenetic tumor suppressor in ovarian cancer. CD79A and CD79B can be found in 8% to 12% of WM patients.^1^ Both are components of the B-cell receptor (BCR) pathway, and can form heterodimers with each other. The CD79A/B heterodimer associates with the immunoglobulin heavy chain required for cell surface expression of BCR, and BCR induced signaling. Activating mutations in the immunotyrosine-based activation motif (ITAM) of CD79A and CD79B have been reported in ABC DLBCL, and trigger SYK, PLCγ2, and BTK.

### Mutations in MYD88 wild-type WM

A small number of WM patients (5%) lack mutations in MYD88, and their disease course is marked by increased risk of disease transformation and shorter overall survival.^13^ Moreover, these patients show little activity to ibrutinib.^3^ These findings point to fundamental differences in underlying genomics. Whole exome sequencing identified somatic mutations in MYD88 wild-type WM patients that are predicted to trigger NFKB (TBL1XR1, PTPN13, MALT1, BCL10, NFKB1, NFKB2, NFKBIB, NFKBIZ, and UDRL1F), impart epigenomic dysregulation (KMT2D, KMT2C, KDM6A), or impair DNA damage repair (TP53, ATM, and TRRAP).^10▪^ Predicted NFKB activating mutations were downstream of BTK, and many overlapped with somatic mutations found in DLBCL.^10▪^

### Copy number alterations

Copy number alterations are common in MYD88 mutated WM patients, and involve both chromosome 6q, and non-chromosome 6q regions.^1^ In chromosome 6q, loss of genes that modulate NFkB activity (TNFAIP3, HIVEP2), BCL2 (BCLAF1), apoptosis (FOXO3), BTK (IBTK), plasma cell differentiation (PRDM1) and ARID1B occur.^12^ Non-chromosome 6q genes that are commonly deleted include ETV6, a transcription repressor; BTG1, that often is deleted in DLBCL, and associated with glucocorticoid resistance in acute lymphocytic leukemia; as well as LYN, a kinase that regulates BCR signaling.^1^ PRDM2 and TOP1 that participate in TP53-related signaling are also deleted in many WM patients. In contrast to MYD88 mutated WM, recurring copy number alterations are rare in MYD88 wild-type WM, including loss of chromosome 6q.^10▪^

### Clinical perspectives

At present, MYD88 mutational status can help in the diagnosis, and prognosis of WM patients.^1^ Those lacking mutated MYD88 by allele-specific PCR analysis for the L265P mutation should be investigated for non-L265P mutations by Sanger sequencing. Patients with suspected WM and wild-type MYD88 should be excluded for other diagnoses including IGM myeloma.^13^ Patients wild-type WM show increased incidence of disease transformation, and can be more closely monitored.^13^ MYD88 mutation status can also serve as an important predictive marker for use of ibrutinib (and possibly other BTK-inhibitors) in WM patients.^3^ Patients with wild-type MYD88 are not very responsive to ibrutinib, and therefore its use should be reserved for those WM patients with mutated MYD88.^3^ CXCR4 mutations are associated with delayed response, lower rates of deeper responses including very good partial response attainment, and shorter progression-free survival.^16▪^ The former is important to consider in patients in whom rapid responses are required for disease control.^1^

## Future perspectives

The discovery of recurring somatic mutations in MYD88, CXCR4, ARID1A, and CD79B mutations in WM offers important new insights into the pathogenesis, prognostication and therapeutic development for WM. The latter includes agents that target key pro-survival signaling in the MYD88 pathways including inhibitors of BTK, IRAK, and HCK. The BTK inhibitors ibrutinib, acalabrutinib and zanubrutinib have shown remarkable activity in WM, and ibrutinib is now approved in the US and Europe, as well as other countries for the treatment of symptomatic WM.^17▪^,^18^,^19^ Selective inhibitors targeting IRAK and HCK are also in pre-clinical and/or early stage clinical development. CXCR4 represents an important target in WM, and a clinical trial assessing the impact of the CXCR4 inhibitor ulocuplomab with ibrutinib in CXCR4 mutated WM patients (NCT03225716) is ongoing with encouraging early findings.^20^ Targeting CD79B signaling through use of SYK inhibitors also appears feasible based on preclinical studies, which show the potential for synergistic interactions with ibrutinib, and may be suitable for those patients with CD79B mutations.^21^ The genomic findings, therefore, lay the foundation for targeted drug development, and the potential for a personalized medicine approach to WM.
